# Schizophrenia and neighbourhood deprivation

**DOI:** 10.1038/tp.2016.244

**Published:** 2016-12-13

**Authors:** S H Gage, G Davey Smith, M R Munafò

**Affiliations:** 1 MRC Integrative Epidemiology Unit, University of Bristol, Bristol, UK; 2 School of Experimental Psychology, University of Bristol, Bristol, UK

Sariaslan *et al.*^1^ found that genetic risk for schizophrenia predicts neighbourhood deprivation, which they interpret as evidence against environmental causes for this association. However, their findings are consistent with a causal association between schizophrenia and later social deprivation.

The prevalence of schizophrenia is strongly socially patterned, being higher in more deprived sections of society. One possible explanation for this is that deteriorating mental state results in lower social position (social drift). Another is that social position is a contributing factor for mental illness (social causation). Sariaslan *et al.*^1^ used a polygenic risk score for schizophreni, and sibling and twin designs to capture genetic variation, to examine the hypothesis that patients with psychosis experience downward social mobility as a consequence of their symptoms. They found that both being a sibling of a schizophrenia proband and schizophrenia risk captured by the genetic risk score predicted neighbourhood deprivation, and concluded that this provides evidence against environmental causes for this association, and in support of genetic influences. They argue for a genetic selection interpretation, whereby genetic liability for schizophrenia predicts subsequent social position, reflected in neighbourhood deprivation.

These findings are also consistent with a causal association between behaviours seen in those at high risk of schizophrenia and subsequent social position. Genetic risk score analysis is akin to a Mendelian randomization design,^2^ whereby genetic variants are used as a proxy for an exposure of interest, to assess the unconfounded association between exposure and outcome. A genetic variant or risk score robustly associated with an exposure of interest could serve as an unconfounded proxy for behaviours of the kind seen among those at high risk of schizophrenia, as we inherit our genetic information independently of environmental factors, and each gene is inherited largely independently of other genes.

Schizophrenia is a rare outcome (and if effects on social mobility are only seen among those who develop schizophrenia, this could not generate an effect of the magnitude seen), but it is plausible that a higher genetic risk score could predict subclinical psychotic-like symptoms in the healthy population. Although evidence is limited, genetic risk for schizophrenia predicts dropout from the Avon Longitudinal Study of Parents and Children,^3^ which could reflect, for example, a more chaotic lifestyle. These subclinical psychotic-like experiences could influence the risk of social drift, without necessarily reaching the threshold for a clinical diagnosis. Indeed, in the same study, polygenic risk for schizophrenia predicts childhood psychopathy.^4^ There is a positive association between risk score and psychotic experiences at the most liberal *P*-value threshold, while at the most stringent threshold this seems to reverse. This could be an example of selection bias, where those at the highest biological risk of schizophrenia are more likely to have left the study,^3^ so that the individuals with the highest biological risk who remain in the study may be unusually healthy. Psychotic-like experiences or unusual behaviour and experiences might therefore plausibly negatively impact upon social position and lead to downward drift. A recent twin study suggested that positive psychotic experiences are associated with stressful life events in adolescence, and that genetic influences explain most of this association.^5^ This suggests a possible mechanism whereby genetic risk for schizophrenia could impact on the occurrence of stressful life events, and in turn social drift.

The distinction between biological and mediated pleiotropy is critical here.^6^ Biological (or horizontal) pleiotropy refers to a genetic variant influencing multiple phenotypes separately, via distinct biological pathways (Figure 1a). Mediated (or vertical) pleiotropy refers to a genetic variant influencing multiple outcomes via one biological pathway (for example, genetic effects on smoking behaviour also influencing lung cancer outcomes, via smoking)^7^ (Figure 1b). In the case of mediated pleiotropy, other influences on the upstream phenotype (for example, smoking) would influence the downstream outcome (for example, lung cancer). Genetic studies can therefore provide insights into the modifiable causes of health and social outcomes.^8^

Sariaslan *et al.* interpret their bivariate quantitative genetic twin models as indicating that genetic influences account for the association between schizophrenia and neighbourhood deprivation rather than environmental factors, and argue that this is evidence against an environmental cause of social drift associated with schizophrenia. However, these findings are consistent with a causal relationship between behaviours related to schizophrenia risk and neighbourhood deprivation. Where the same genetic variants appear to predict two outcomes, this could suggest shared genetic aetiology, as the authors conclude, but it could also reflect a mediated causal relationship.^8^

In our opinion, further research to determine whether genetic risk for schizophrenia influences social position via behaviours associated with that risk is warranted. The crucial point is that, if correct, our interpretation implies that it is behaviours present in those at high risk of schizophrenia that are causally related to social position. This could represent a modifiable target for intervention—since such behavioural patterns are influenced by factors other than genetics—and provide crucial insights to policy makers and clinicians. Once genetic results are interpreted through the lens of Mendelian randomization, they are no longer informative only about genetic influences, but also about modifiable influences. Since the genomewide association study used by Sariaslan and colleagues to generate a genetic risk score for schizophrenia was published, a larger study has identified 108 genetic variants at genomewide significance, explaining substantially more of the variation in schizophrenia risk.^9^ This could be utilized to explore how high genetic risk of schizophrenia impacts a non-clinical sample with higher power, and explore these relationships in more detail, to untangle causality.

## Figures and Tables

**Figure 1:**
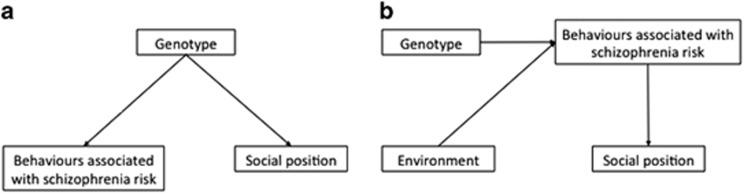
Directed acyclical graphs showing (**a**) shared genetic architecture (biological or horizontal pleiotropy), where genotype influences two separate phenotypes, and (**b**) mediated (or vertical) pleiotropy, where genotype impacts on one phenotype, which itself causally affects the second phenotype. In this instance, environmental factors can also impact on the first phenotype, meaning that there is the possibility for targeted interventions that can minimize the first phenotype (in this case behaviours associated with schizophrenia risk), which would also have a downstream effect on the second (social position).

## Notes

This work was supported by the Medical Research Council Integrative Epidemiology Unit at the University of Bristol (MC_UU_12013/6). The authors declare no conflict of interest.
